# Developing Collaborative Practices in Co-Located Health and Social Services: An Observational Study of a Multidisciplinary Development Group

**DOI:** 10.5334/ijic.8939

**Published:** 2025-09-22

**Authors:** Kaisa Pasanen

**Affiliations:** 1 Faculty of Social Sciences, University of Helsinki, Finland

**Keywords:** integrated care, health care, social services, collaboration, adults

## Abstract

**Introduction:**

Despite ongoing efforts to integrate health and social services, achieving integration in front-line practice remains challenging. This study explored collaboration in a multidisciplinary development group aimed at improving collaboration in one co-located health and social services centre in Helsinki, Finland. Drawing on the notion of knowledge practices as means for solving complex issues, the study analysed how collaboration and co-development are enacted within the group.

**Description:**

Observational data collected from 11 development workshops were analysed using an abductive approach. The analysis identified three modes of collaboration: 1) one-sided knowledge sharing, 2) collaborative knowledge sharing, and 3) collaborative knowledge creation.

**Discussion:**

Shared concepts and a new multidisciplinary service process supported collaborative modes for working, while some facilitation practices seemed to hinder them. The findings revealed a gap between the ideal of bottom-up development and the organisational conditions influencing it. A clear mandate and purposeful facilitation are critical to achieving the intended goals of such development initiatives.

**Conclusion:**

Stronger theoretical foundations and explicit theories of change could enhance development efforts. This study identified theoretical concepts that illuminate collaboration as a contextually shaped set of social practices. These insights can contribute to the design and facilitation of practice-based development efforts.

## Introduction

Challenges such as fragmented service systems, demographic change, and budget constraints have driven health and social service organisations to seek new approaches to delivering services – particularly for people with multidisciplinary service use. Integrated care is a widely adopted approach that stresses principles such as continuity of care, multidisciplinary collaboration, and service user participation [1,2,3]. However, implementing these principles in everyday practice has proven to be challenging [4,5], and there is a persistent gap in collaboration between health and social services in particular [6,7,8]. Previous studies indicate that a strong focus on the structural integration of services at the expense of collaboration in practice may undermine the results of integrated care initiatives [9]. In addition to structural changes, achieving change in everyday practice requires attention to the mechanisms that influence collaboration in front-line work [7,9].

Multidisciplinary working is generally regarded as an integral element of integrated care, and extensive research exists on its facilitators and barriers. Several reviews have identified shared goals, clarity in professional roles and responsibilities, communication, and information sharing as central factors that enhance or, if not successfully realised, hinder collaboration [4,10,11,12]. Previous studies suggest that purposive, practice-led learning and development activities could be key elements in overcoming the identified barriers to collaboration [13,14]. However, there is limited research on such initiatives and how collaboration is enacted within them – especially in the context of health and social service collaboration.

The present article reports findings from a study on a new development intervention that focused on improving multidisciplinary collaboration in a newly opened health and social services centre in Helsinki, Finland. Using observational data, the aim is to explore how this intervention unfolds in practice, and what can be learned from collaborative processes for the design and implementation of practice-led development efforts.

In Finland, health and social services are highly integrated at the organisational level, as both are organised by regional authorities. However, service organisations face similar challenges in achieving integration in practice as other health and social care providers worldwide [7,15]. To address challenges related to coordination and continuity of services, the City of Helsinki has established multidisciplinary health and social services centres (HSSCs) that provide services primarily for working-age adults. These services include primary and oral health care services, psychiatric and substance misuse services, adult social services, services for immigrants, and services for people with disabilities. This strategic initiative aims to improve access to services, user experience, and, ultimately, effectiveness of services. Furthermore, it aims to tackle issues related to health and wellbeing inequalities by providing highly integrated health and social services to people with high or multidisciplinary service use. Co-location of services is expected to enhance multidisciplinary working and the development of new collaborative practices.

To improve health and social service collaboration for adults with high or multidisciplinary service use, a new multidisciplinary service process was introduced as part of the development of the HSSCs. The service process includes elements of the chronic care model [16] but aims to address both health and social service needs. Central elements of the service process are: 1) a named contact person for each client, 2) multidisciplinary service needs assessments and service plans, 3) enhanced client participation, and 4) self-management support. Additionally, a multidisciplinary development group involving professionals from different services was formed in each HSSC to support the implementation of the service process. The present study focuses on the work of one of these groups.

Following Hughes et al. [17], integrated care is analysed as a set of social processes and practices emerging from complex contexts. Theoretically, the study draws from pragmatist philosophy and the notion of knowledge practices [18] as means to address complex and open-ended issues. This perspective focuses on practical actions in multidisciplinary working: how practitioners work in situations that involve shifting between routine procedures and negotiating solutions to complex issues, how practices develop, and how contextual factors shape them [18,19]. The specific research question is: *how are collaboration and co-development enacted within a multidisciplinary development group in a co-located health and social services setting?*

## Research methods

### Study design

The present study is a part of a larger case study [20] that explores knowledge practices in one purposefully selected HSSC that was opened in 2018. The research site is one of the first large-scale health and social services centres in Finland with purpose-built facilities and targeted organisational supports designed to enhance collaboration. As such, it provides an information rich case that can offer insights into the critical components of a complex intervention and extend understanding of real-life multidisciplinary collaboration [20,21]. Following a practice research approach [22], the study was designed with practice stakeholders. This involved identifying relevant research questions and planning data collection with the developers of the HSSC and a group of experts by experience. Observation and audio recording were chosen as methods for data collection to gain insight into real-life multidisciplinary interaction and dynamics within the new development group [21].

### Theoretical framework

The study is underpinned by pragmatist philosophy, which seeks to understand ideas in relation to their consequences [23]. Inquiry is viewed as a dynamic problem-solving process aimed at producing practice relevant knowledge in real-life contexts [24,25]. Thus, pragmatist research provides a particularly suitable starting point for studying how the new development-oriented initiative unfolds in its context. In particular, Peirce’s [23] ideas of abductive reasoning and Dewey’s [26] view of concepts and theories as hypotheses refined through empirical inquiry have informed the study.

The present study analyses multidisciplinary working as knowledge work [27], where knowledge from different disciplines and perspectives is applied to discover practical solutions that support collaboration. Knowledge practices – also referred to as epistemic practices – are practices for using knowledge to address complex and open-ended issues [18]. Such issues cannot be resolved solely by relying on routines or discipline-specific ways of working. For example, planning care and support for a person with multidisciplinary service use requires working together and adapting knowledge to the client’s individual situation within a specific context [19,28]. This kind of work involves knowledge practices that include: 1) sharing knowledge across disciplinary boundaries, 2) generating and accumulating knowledge, and 3) applying knowledge to solve complex issues [18,29].

Knowledge practices form around shared objects of knowledge – that is, objects of collaborative work that are examined, developed, and utilised together [30,31]. In integrated care, such objects can include, for example, clinical procedures, practice models, or service plans. Knowledge practices that form around these objects can be collaborative or one-sided in nature depending on how the practitioners engage in working together. Collaborative practices are shaped by shared language, ideas, or concrete tools, such as technology or standardised forms [32]. In one-sided practices, the language and practices of one discipline or, for example, organisational guidelines overpower the expertise of others [33]. Achieving integration requires identifying mechanisms that support collaborative practices which draw on diverse forms of knowledge.

In this study, the notion of knowledge practices is employed as an analytical framework to understand, first, the processes involved when practitioners aim to find solutions to working with complex service needs. Second, it is applied to identify contextual elements that influence multidisciplinary working.

### Intervention and participants

To support the implementation of the new service process, develop working practices related to the process, and co-create solutions to challenges identified in multidisciplinary working, a new development intervention was designed by the HSSC project team. The intervention comprised facilitated and structured monthly workshops of a multidisciplinary development group involving professionals from different services within the HSSC. A group of experts by experience contributed to planning the themes of the workshops from a service user perspective.

The group involved 20 health and social service professionals comprising social workers (SW, n = 4); social services counsellors (SsC, n = 4); mental health (MhN, n = 4), substance misuse (SmN, n = 1), primary care (PcN, n = 1), and dental (n = 2) nurses; an occupational therapist; a physiotherapist; a general practitioner (GP); and a facilitator (F) working as a coordinator in the HSSC. The facilitator was part of the team responsible for designing the intervention. The participants represented primary and oral health care, adult social services, services for people with disabilities, services for immigrants, psychiatric and substance misuse services, and rehabilitation. One of the workshops, where the main theme was user experience, involved three trained experts by experience (EbE). Furthermore, three workshops involved a student observing the work as a part of their practice placement.

Each workshop was based on a theme concerning clients with multidisciplinary service use, such as mental health, substance misuse, homelessness, or long-term or complex physical health issues. Between workshops, the participants trialled suggestions for improving multidisciplinary working that emerged during the sessions. The duration of each workshop was two hours, which included an introduction to the topic of the workshop and discussion on issues and possible solutions related to multidisciplinary working concerning the topic. In the first five workshops, case vignettes made by the HSSC project team were utilised to elicit discussion. The following workshops were based only on the introduction and a general discussion on how to help clients with service needs related to the topic of the workshop. The final workshop in October 2020 comprised evaluation of the workshop process. Six of the workshops were held in person at the HSSC and five remotely via Microsoft Teams after the COVID-19 outbreak in March 2020. The implications of the pandemic are discussed in later sections.

### Procedure

The study followed the ethical guidelines of the Finnish National Board on Research Ethics [34] and received a research permit from the City of Helsinki Social Services and Health Care Division. Following the national guidelines for ethical review in the human sciences [35], further ethical approval was not required, as participation was based on informed consent and was not considered to induce harm to the participants. Furthermore, the workshops did not involve discussing patient or client data or details of real-life client cases. The participants were informed of the study via email before the first workshop and in person at the beginning of the first workshop where written consent was obtained from each participant. The expert by experience and student participants, who each participated in only one workshop, were informed at the beginning of these workshops and their consent was obtained at the same time.

The data were collected from 11 workshops of the multidisciplinary development group during its first year of work between October 2019 and October 2020. The workshops were audio-recorded by the author. Field notes were made on general observations from the workshops, such as the atmosphere and seating arrangements. In addition, written comments from the Microsoft Teams chat were collected during the remote meetings. The sections of the audio recordings involving discussions were professionally transcribed. The transcribed sections varied from 24 to 94 minutes per workshop and comprised 8.5 hours of data altogether.

The author participated in the workshops and observed the discussions but only took part in discussions during introduction rounds and informal encounters after the workshops. The author played a more participatory role in the first workshop in introducing the study and presenting a short introduction to multidisciplinary work from a research perspective. In the final workshop, the author was asked to reflect on their experiences and observations of the workshop process as a researcher.

### Analysis

**Figure 1:**
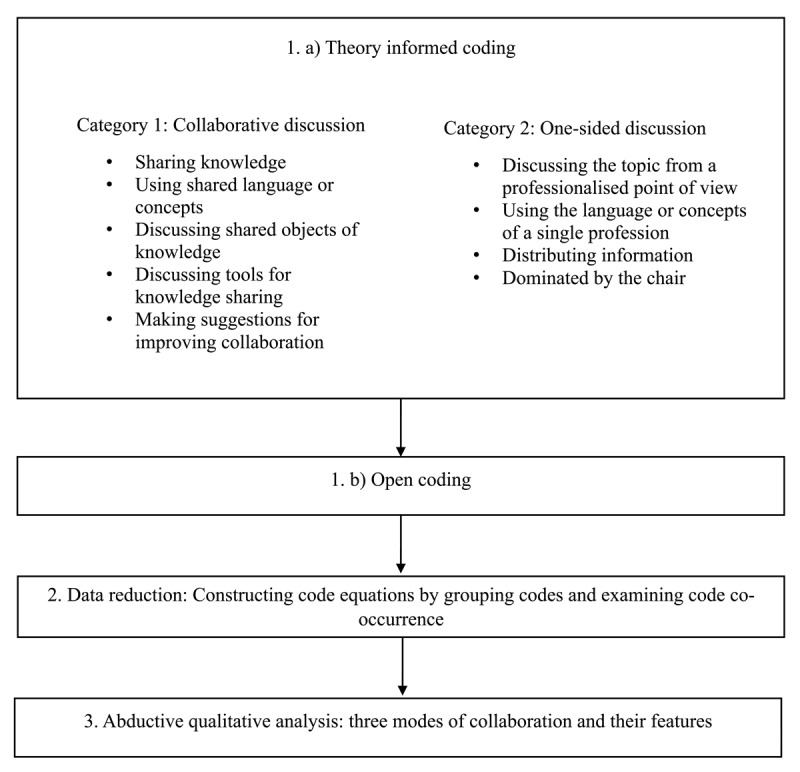
Stages of analysis, and categories used in theory-informed coding.

The data were analysed following Vila-Henninger et al.’s [36] approach to abductive analysis [37], which involves three stages: 1) generating an abductive codebook based on initial deductive coding followed by open coding, 2) data reduction through code equations and, 3) in-depth abductive qualitative analysis (Figure 1). ATLAS.ti 9 software was used to conduct the analysis. The analysis focused on the transcribed workshop discussions.

Drawing on the understanding of knowledge practices as collaborative or one-sided in nature, the initial coding included identifying, first, segments where participants engaged in sharing knowledge, using shared language, discussing shared objects of knowledge, such as processes within the HSSC, or making suggestions for improving collaboration. This category was named *collaborative discussion*. Second, segments where professionals discussed the topics only from their own professional point of view, used the concepts of a single profession, or distributed information without an evident aim to discuss it with others were identified. The second category also included segments where the discussion was dominated by the chair. This category was named *one-sided discussion*. In the second stage of the analysis, the data were coded using open coding. The codes were then refined, and the full data were coded using the finalised coding frame.

The second stage of the analysis involved generating code equations by combining deductive and inductive codes. The rationale here was to identify, how the inductive codes relate to theoretical constructs, and develop more nuanced themes [36]. Code co-occurrence and network tools in ATLAS.ti were employed to examine connections between codes. This stage resulted in three code equations, i.e. themes, that were analysed in detail in the final stage of the process. This analysis involved moving back and forth between empirical data and theoretical constructs to look for potential explanations for empirical observations and refine the theoretical framework [36,37].

The analysis revealed that the initial idea of the development group as a forum for developing multidisciplinary knowledge practices did not account for all empirical observations. Thus, the analysis shifted to theorising the group itself as a knowledge practice. This involved additional open coding and revisiting the theoretical framework to focus on the characteristics of the group as a knowledge practice. Here, the concept of knowledge creation [38] was incorporated into the analysis to provide a more nuanced understanding of collaboration within the group. The analysis and primary results were discussed with practice stakeholders to verify the interpretation of the data.

## Results

The analysis identified three distinct modes of collaboration within the development group: 1) one-sided knowledge sharing, 2) collaborative knowledge sharing, and 3) collaborative knowledge creation. The three modes of collaboration, their features, and the suggestions made for improving collaboration are summarised in Table 1.

**Table 1:** Modes of collaboration in the multidisciplinary development group, their features, and suggestions for improving collaboration.


MODE OF COLLABORATION	PROMPT FOR DISCUSSION	FEATURES OF THE DISCUSSION	SUGGESTIONS FOR IMPROVING COLLABORATION

**One-sided knowledge sharing**	Identifying inaccuracies or errors in the case vignettes	Detailed discussion on the case vignettes Using the language and concepts of a single profession Focusing on details of care from a single professional perspective	No suggestions for improving collaboration

**Collaborative knowledge sharing**	General discussion on the case vignettes or theme of the workshop Questions to other professionals about their work and practices	Identifying needs for changes in practice Using shared language and concepts Discussing tools for knowledge sharing Reflecting on an individual professional’s contribution to a specific case or problem	Concrete tools to improve knowledge sharing: technological tools, lists of consultation numbers, naming contact persons for consultations

**Collaborative knowledge creation**	Discussing shared objects of knowledge (e.g. the multidisciplinary service process, service and care plans)	Identifying needs for changes in practice Using shared language and concepts Negotiating solutions Reinforcing other participants’ arguments Adding perspectives to the discussion	Multidisciplinary consultation meetings Multidisciplinary meetings with clients present Shared service or care plans


Overall, the workshop discussions focused, first, on challenges in supporting clients with multidisciplinary service use, including institutional barriers to collaboration and client-related factors such as the complexity of service needs, motivation, and engagement in service processes. Second, the group discussed the implementation of the newly introduced service process and how it should be implemented in practice. The differences between live and virtual workshops were considered as a part of the analysis. Although interaction in the virtual workshops was slightly more rigid compared to live workshops, the modes of collaboration were similar. Rather than the virtual environment, the modes of collaboration were influenced by the same elements as the live workshops. The following sections describe each mode of collaboration in detail.

### One-sided knowledge sharing

Considering the development groups’ objective of enhancing multidisciplinary working, the least productive mode of collaboration was one-sided knowledge sharing, where participants distributed information without an evident intention to discuss it with others. However, this mode was also the least evident in the workshops. One-sided knowledge sharing was often prompted by the case vignettes utilised in the workshops when participants identified errors or deficiencies in the vignettes. This, in turn, led to detailed discussions on what assessments, tests, or clinical examinations should be conducted before deciding on a further care or service plan. Overall, these discussions were characterised by talking about details of client care from a single professional perspective rather than stepping back to view the issue on a more general level.

The following data excerpt is an example of one-sided knowledge sharing, where the participants get stuck in discussing the details of a case vignette of a person with multiple service needs who has a care coordinator from a third-sector service provider but who has not shown up to appointments in the HSSC. The participants perceived the vignette as lacking key information about the case, which led to detailed discussion on the specific case instead of how to help clients navigate fragmented services.

“SW: I see the problem in adult social work, as a social worker, that all of these clients could fall under our service needs assessment [ ] But to me, it still feels very fragmented. Where do I even start to put together the service package for a person? The multidisciplinary support.

MhN 1: In my opinion, the care coordinator is the gold nugget here – that’s where it all begins. That day-to-day support: they see the everyday reality – the depression, the mental well-being, the physical condition. What does it mean? That case vignette doesn’t explain any of it.

MhN 2: An unstable personality isn’t a disease, it’s a disorder. A panic attack is a diagnosis. What is it, really? I got stuck on this little thing right away. But if you know that this might be a client who doesn’t show up – if it’s a typical unstable personality – that already tells you that the contact person is really important. [ ]

GP: And with that kind of patient, the summary [in the patient record system] is often really helpful. If you go through all the records – what’s there? It might say he’s fallen, had a head CT last week, and so on. So, the summary kind of pulls everything together.”

As in this excerpt, one-sided knowledge sharing was often dominated by health care perspectives and jargon, such as talking about diagnoses, clinical examinations, or health care documentation, which impeded joint work on improving practices. These discussions did not result in suggestions for improving multidisciplinary working but rather stated a specific point on the case discussed. However, one of these discussions resulted in disagreement between the participants, which, in turn, led to a collaborative discussion on what could be done differently.

This mode was partly characterised by the facilitator taking an authoritative role in controlling discussions and the participants’ contributions. In the next excerpt, the facilitator interrupted a discussion on one of the case vignettes to explain their perspective:

“F: Let’s get back to this point. So, in this case it is very important that regardless of where you work – this helps primary health care’s work a lot – if you’ve already found out in advance which health centre the client has used, and whether a specific nurse or doctor has already been assigned, because these clients often have a lot of information. Even though, from the social work perspective, it was mentioned that they don’t always remember it.”

After this, the facilitator continued to explain what each professional should do in the case and what could be the underlying issue behind the client’s health care needs. Like in this excerpt, the facilitator could overpower participants’ perceptions by stating their opinion on the topic of discussion and, thus, prevent participants from making suggestions However, the facilitator could also play a role in guiding the discussion towards a collaborative mode, as described in the next section.

### Collaborative knowledge sharing

The primary mode of collaboration in the workshops was collaborative knowledge sharing, where participants utilised shared language and concepts to reflect on how each professional could contribute to a specific case or problem. This mode was likely to result in concrete suggestions of tools to improve knowledge sharing, such as lists of consultation numbers or contact persons. The discussions were prompted by the case vignettes when they were approached on a general level or the participants’ questions to each other about their work. However, in this mode of collaboration, the participants were not likely to cross professional boundaries or suggest new working practices.

Collaboration in this mode was mediated by concepts that had initially been introduced to describe service processes and professional roles within the HSSC. Although the use of these concepts was organisationally driven, the participants adopted them as a shared language to discuss helping clients with multidisciplinary service use. For example, in the next excerpt, a social services counsellor from adult social services uses shared concepts (MSU process and contact person) to discuss the value of the new service process:

“SSc: When we place someone into housing services, the MSU [multidisciplinary service use] process is crucial. It’s absolutely crucial that we know the client’s network [of professionals], along with the contact person if a person is placed directly from the hospital, a child protection facility, or even from home – like an elderly couple who hasn’t been involved with any services for ten years – until suddenly there’s an acute need, and now they have to enter housing services.”

This excerpt was followed by a discussion on improving health and social service collaboration for people in housing services. The group concluded that each housing unit should have named contact persons from adult social work and primary health care, and that these names should be added to a list of consultation contacts within the HSSC. Using shared language instead of professionalised jargon enabled all participants to engage in such discussions. In addition, some health care professionals were remarkably active in asking questions about social services, as many of them felt that they had limited knowledge on them. This promoted knowledge sharing between participants which, in turn, prompted discussions on how to improve knowledge sharing beyond the workshops.

In contrast to one-sided knowledge sharing, the group’s facilitator played a positive role in promoting this mode of collaboration. They ensured that all views were taken into account, encouraged everyone to participate, and directed the discussions towards considering different aspects of the problem at hand. Furthermore, the facilitator encouraged making concrete suggestions to develop multidisciplinary working. In the following excerpt, the participants discuss a case vignette of a person with excessive alcohol use and multiple visits to urgent health services. The facilitator encourages discussion, and the participants engage from their own professional perspective but use shared concepts to discuss the case:

“F: What do the others think? Give some comments – don’t worry about saying anything wrong. Let’s think about this together.

MhN: From psychiatry, we would probably do a home visit.

SmN: In my case, she would be a client in substance misuse services. I would want a social worker as the contact person, or an occupational therapist, someone who can assess the functional capacity. But of course, I would also involve primary health care – I might walk down the corridor [to a primary care professional] or call our consultation number. So, an assessment of health needs, and then a care plan could be created within primary health care.

F: Any other comments? Comment freely – let’s discuss this together.

SW: I’d ask about how she’s coping at home, because that assessment is missing here, and then contact primary health care.”

This passage was followed by further discussion on how each professional would assess the client’s service needs and talk about them with the client as well as identifying issues related to knowledge sharing between services.

In this mode of collaboration, suggestions for developing multidisciplinary working were mostly focused on improving communication, consultation, and information sharing between professionals. Insufficient tools for communication between professionals and services in the HSSC were perceived as a significant barrier to collaboration. This was related, first, to the lack of shared information systems and, second, to the variety of services and working practices within the HSSC. This led the discussions to focus on drawing up and sharing this information instead of practices for collaborative working.

### Collaborative knowledge creation

The third mode, collaborative knowledge creation, was most likely to result in suggestions for new multidisciplinary practices. In contrast to knowledge sharing, knowledge creation was characterised by attempts to translate knowledge into concrete changes in practice. In this mode, the participants engaged in negotiating a shared understanding of the purpose and use of the new service process and making suggestions to improve multidisciplinary working. This mode of collaboration was less evident in the workshops compared to knowledge sharing, although the primary objective of the development group was to foster this kind of collaborative working.

Like collaborative knowledge sharing, collaborative knowledge creation was characterised by using shared language but also active sense-making of the new multidisciplinary service process. In the discussions, this was displayed as reinforcing each other’s arguments, adding perspectives to the discussion, and jointly negotiating solutions to improve multidisciplinary working. In contrast to knowledge sharing, this mode resulted more often in suggestions of practices for multidisciplinary working in complex client cases, such as case meetings with clients present, shared service plans, and regular consultation meetings. In the following excerpt, the participants identified an issue related to fragmented service processes based on one of the case vignettes:

“SSc: In this case, I feel like it really shows that the client has a lot of services but doesn’t feel like they’re receiving any support that actually helps them. There might be separate plans made in adult social work that include certain services, and plans in psychiatric outpatient care or primary health care. All these need to be brought together.

EbE: Exactly. If everyone makes different plans, then as a client, I’d be completely confused.

GP: Overwhelmed.

EbE: One person says one thing, the doctor says something else, and another person explains it differently.

GP: Yes, and the connections between the separate plans is also unclear.

SSc: Right.

EbE: So, all those plans absolutely need to be combined.”

This discussion concluded with a suggestion of multidisciplinary appointments where different perspectives could be discussed collaboratively to draft a client-centred service plan. Additionally, the group suggested that trained experts by experience could be involved in appointments with clients who experience difficulty in committing to services.

The multidisciplinary service process, which had initially been a top-down initiative, developed into a shared object of knowledge that engaged practitioners in collaborative working to make this process function in practice. Professionals from different disciplinary backgrounds valued different elements of the service process. For example, social service professionals perceived closer collaboration with health care as a key element of the process, while primary care professionals valued enhancing continuity of care. Despite these differences and the top-down nature of the initiative, the service process seemed to provide enough common ground to promote collaboration.

A central suggestion that emerged from this mode of collaboration was a multidisciplinary consultation meeting, developed in response to practitioners’ needs for support in complex client cases. In the next excerpt, the participants discuss the consultation meeting following a discussion on improving consultation practices and whether a list of consultation numbers or a weekly consultation meeting would be more beneficial. The discussion is characterised by joint attempts to negotiate how the consultation meeting could work:

“SsC: I don’t know if I’m the only one, and I’m flexible about this thought. The idea sounds good, but I still question the feasibility of it, that someone from every unit can participate every week. [ ] I might not be able to share that responsibility as much as many others here. [ ]

PcN: But just knowing it’s possible.

GP: There’s no need from our side, but we’ve benefited tremendously when the social worker from substance misuse services has participated. [ ] So just having one social services professional there, that helps us. It could be anyone. [ ]

SsC: It’s nice, definitely nice for us to know that if I had an issue, I could come there. But it’s not reciprocal. I mean, there should be input the other way too – so people know there’s someone from housing support. Maybe that could happen once a month.

GP: You’re probably right that there’s no need for someone from every unit to participate every Wednesday. [ ] We can’t expect someone from every team to be there. No. But what matters is that the information reaches the other services, and that we understand each other’s practices.”

In the following workshops, the participants returned to this topic several times. During the workshop process, the consultation meeting developed into a weekly virtual opportunity to consult other professionals and learn from others. This practice was later perceived as one of the key elements promoting multidisciplinary working.

Despite the groups’ intended focus on developing working practices, collaborative knowledge creation often seemed to be impeded by institutional and organisational boundaries for service delivery, such as criteria for accessing services, referral practices, and time constraints. Changing these practices were perceived as decisions to be made at the organisational level and, therefore, out of the scope of the practitioner-level development group. For example, one of the participants described the lack of in-home support for working-age adults:

“SSc: For example, we social services counsellors currently don’t have the resources for this kind of work. The clients at psychiatric outpatient clinics, the mental health clients – they do [have access], but we can’t influence that.”

Although the participants agreed that such support would be greatly needed, they could not work out a solution as decisions on resources and criteria for accessing services are made elsewhere.

Next, the article turns to discussing the findings and their implications for designing and implementing similar development interventions.

## Discussion

The aim of this study was to explore, how a new development intervention focused on improving multidisciplinary working in co-located health and social services unfolds in practice, and what can be learned from collaborative processes within the group for such initiatives beyond this specific context. By applying concepts from practice theory and knowledge management, the current study identified three distinct modes of collaboration: one-sided knowledge sharing, collaborative knowledge sharing, and collaborative knowledge creation.

The results show that collaboration within the development group was mostly centred around knowledge sharing although the primary aim of the group was to co-create solutions related to the implementation of a new service process. Despite co-location, the participants experienced having a limited understanding of other professionals’ work or who to contact in different situations. As the development process took place during the early stages of the HSSC, there may not yet have been enough common ground to achieve the intended aims. However, given that shared understanding is an important prerequisite for collaboration [14,39], both one-sided and collaborative knowledge sharing could be essential steps in moving towards co-creating solutions for service delivery. Productive collaboration may also require moving between the different modes of collaboration depending on the problem at hand. While complex issues call for co-creation and adaptive knowledge work, well-defined problems can be addressed through routines and established professional roles [40].

During the workshops, the new multidisciplinary service process formed into a shared object of knowledge [18,30] that engaged professionals in working together and bridged gaps between disciplines. This observation follows previous research on boundary objects that hold integrating potential [41,42,43], provided that professionals are engaged in making them work in practice. Surprisingly, the case vignettes intended to support co-creation did not seem to function as boundary objects but instead drew attention to flaws in the vignettes, especially from a medical perspective. As the new service process was grounded in the chronic care model it may have contributed to a strong focus on health risks in some of the discussions. Fleming et al. [44] note that boundary objects grounded in medical models may risk medicalising social services, as they can also disguise tensions among stakeholders [see also, 41]. Applying such models to enhance collaboration between health and social services warrants further research on their influence on direct practice with clients.

Another challenge related to the facilitation of the group was the facilitator’s ambivalent role, which has also been noted in previous research [45]. While the facilitator could encourage multi-perspective dialogue, they occasionally dominated discussions which, in turn, suppressed other viewpoints. Rather than professional roles, this seemed to be related to the facilitator’s given ‘institutional authority’ [46] to moderate the workshops, and their role as one of the designers of the development intervention.

The findings reveal a gap between the ideal of bottom-up development and the organisational conditions that shape it. The participants were cautious about making direct suggestions for changing practices, as they felt that decisions regarding organisational guidelines, resources or services offered should be made by management. Despite being organisationally driven, the development group seemed to lack a clear mandate. This discrepancy can be understood through the lens of organisational professionalism [47]. Evetts notes that professional work is increasingly influenced by organisational structures and priorities – such as standardisation and performance targets – instead of professional discretion. As a result, practitioners may feel that there is limited space for agency or innovation. This also seemed to manifest in the difficulty in designing practices to solve complex client cases. The participants perceived many service needs to be so complex that, rather than standardised procedures, person-centred services would be best provided through individual tailoring based on professional assessment. However, the organisational logics of service provision seemed to conflict with this kind of logic of care [48,49].

Another potential explanation for the issues related to the facilitation and unclear mandate of the development group is the lack of a clearly outlined theory of change for the intervention. Instead of a systematic process, the development group seemed to be experimental and adaptive in nature. While this kind of approach can be valuable for trialling what works in practice in a new context [25], future initiatives could benefit from stronger theoretical foundations [see also 50,51]. Based on the current study, organisational learning and knowledge management theories could provide helpful concepts for the design of development initiatives. The knowledge practices perspective can illuminate how learning and knowledge management are shaped by collaborative actions embedded in specific organisational contexts [52].

### Limitations and future research

A central limitation of this study is, first, that the analysis was conducted by only one researcher. To limit potential bias, the analysis and interpretation of the results were discussed with senior researchers as well as practitioners in the HSSC. Second, the study observed the work of one development group within a specific context and time. The study was conducted partly during the COVID-19 pandemic, which influenced the work of the group, as its workshops were moved online. Furthermore, some participants were temporarily transferred to pandemic-related tasks. It is possible that, without this change, the group could have formed a more collaborative relationship. However, the group had met several times before the pandemic and established a working relationship, and thus were able to continue their work in virtual workshops. Nonetheless, the processes and results of a similar initiative may be different in other contexts or with different participants. Despite these limitations, the results may help to facilitate practice-led development processes in other contexts, as many service organisations face similar issues in achieving integration in practice.

Finally, the scope of this study was limited to collaboration within the development group. The participants identified challenges in disseminating knowledge to other practitioners and engaging them in collaboration. After the one-year period observed in this study, the group began a second development cycle focusing on issues identified during the first year. Although some participants changed, the foundation for collaboration established in the first year seemed to support further work. Further research is needed to evaluate the long-term outcomes of such initiatives.

## Lessons learned

- Practice-led development interventions hold potential for enhancing shared understanding of collaboration and co-creating collaborative practices within integrated care settings.
- Shared concepts and boundary objects – such as multidisciplinary service processes or pathways – can facilitate collaboration by providing common ground for work.
- Bottom-up development requires a clear mandate and carefully planned facilitation to achieve its intended goals.
- Stronger theoretical foundations and explicit theories of change could enhance development efforts by guiding purposeful action and aligning stakeholder objectives and expectations.

## Conclusion

Practice-led learning and development activities are a potential avenue for improving multidisciplinary collaboration. Understanding how collaboration is enacted within such activities can help in planning and facilitating purposeful interventions. This study identified theoretical concepts that illuminate collaboration as a set of social practices shaped by cultural and material contexts. These insights contribute to more effective design and facilitation of practice-based development efforts. Future research will explore the implications of the development intervention for practice.
